# Magnetically Actuated Hydrogel-Based Microrobotic Structures with X-Ray Visibility for Targeted Drug Delivery in Vascular Environments

**DOI:** 10.3390/mi17101120

**Published:** 2026-09-25

**Authors:** Jiyu Jung, Geonhui Mun, Yoon-Gyung Sung, Kyongsu Lee, Van Du Nguyen, Gwangjun Go, Eunpyo Choi

**Affiliations:** 1 Department of Mechanical Engineering, Chosun University, 309 Pilmun-daero, Dong-gu, Gwangju 61452, Republic of Korea; wjdwldb0426@chosun.ac.kr (J.J.); fa2735@chosun.kr (G.M.); sungyg@chosun.ac.kr (Y.-G.S.); lgs3326@chosun.ac.kr (K.L.); 2 Department of Mechanical Engineering, Sogang University, 35, Baekbeom-ro, Mapo-gu, Seoul 04107, Republic of Korea; nvdu81@gmail.com

**Keywords:** electromagnetic actuation, intravascular navigation, magnetic microrobots, magnetic nanoparticle concentration, targeted drug delivery, X-ray visibility

## Abstract

In recent years, microrobots have attracted attention as minimally invasive therapeutic tools in biomedical applications, as they enable enhanced drug delivery efficiency through direct and localized transport. However, practical realization requires highly precise controllability and real-time responsiveness. In this study, we present a hydrogel-based microrobotic structure precisely and dynamically controlled in real time using externally applied magnetic fields. Chemical, structural, and magnetic characteristics were systematically analyzed, followed by experimental evaluation of targeting performance within a vascular-mimicking model under a phosphate-buffered saline (PBS) environment. Results demonstrate that sufficient visibility in X-ray imaging can be achieved depending on the concentration of embedded magnetic nanoparticles introduced during fabrication. Moreover, stable and real-time targeted navigation of the microrobotic structures was confirmed within specific threshold ranges of magnetic field strength and frequency. By effectively restricting off-target motion, the proposed system offers a minimally invasive medical platform that simultaneously ensures high stability and control precision.

## 1. Introduction

Vascular intervention is a primary therapeutic strategy for cardiovascular and cerebrovascular diseases, providing a minimally invasive approach that promotes patient recovery and reduces surgical burden [1]. Recently, magnetically actuated microrobotic microstructures, controlled by external magnetic fields, have been investigated as an alternative for precise drug delivery and targeted lesion treatment within complex vascular environments [2,3,4,5,6,7,8,9]. Electromagnetic actuation (EMA) systems generate magnetic fields that guide and control these microstructures, enabling navigation in micro-vascular regions that are inaccessible or poorly managed by conventional catheter-based techniques. This approach provides a promising solution to key limitations of current vascular interventions.

Achieving real-time localization and precise control is critical for the clinical translation of magnetically actuated micro-robotic microstructures in complex vascular environments. These microrobots are typically prepared as hydrogel-based structures embedded with magnetic nanoparticles (MNPs) to ensure responsiveness to external magnetic fields. Although X-ray fluoroscopic imaging is the standard intraoperative visualization technique, hydrogel structures are highly X-ray transparent, limiting their visibility and complicating real-time positional identification. Therefore, the X-ray visibility of these microrobotic microstructures depends mainly on the concentration and composition of incorporated additives, such as magnetic nanoparticles.

Previous studies on magnetic alginate-based microstructures have mainly focused on magnetic actuation, drug delivery, and biocompatibility [10,11,12]. Other studies have improved their visibility by adding magnetic nanoparticles (MNPs) or radiopaque materials for diagnostic imaging [13,14,15,16]. However, magnetic actuation and X-ray visibility have generally been investigated separately. Few studies have quantitatively examined how the MNP concentration affects both magnetic responsiveness and X-ray visibility in the same microstructure. In addition, real-time X-ray tracking combined with magnetic guidance toward a selected branch under flowing conditions has not been sufficiently demonstrated.

In this study, MNPs are used as both magnetic components and X-ray contrast agents in alginate-based microstructures. Different MNP concentrations are systematically compared to determine a suitable condition for both magnetic actuation and X-ray visibility. The size of the microstructures can also be controlled by adjusting the centrifugation speed, which allows their delivery through a catheter. Unlike previous studies that mainly demonstrated either magnetic actuation or imaging capability, the present study evaluates catheter-based delivery, real-time X-ray tracking, and magnetic branch-selective movement together in a flowing vascular phantom. In addition, the microstructures are fabricated using an alginate-based hydrogel with biodegradable properties, which may reduce the possibility of long-term retention in the body [17,18].

Based on this background, we present hydrogel-based microrobotic structures for intravascular targeted delivery. The MNPs are uniformly dispersed within the alginate hydrogel matrix, and the fabricated structures are controlled using external magnetic fields generated by an electromagnetic actuation (EMA) system. As illustrated in Figure 1a, the microstructures are introduced into the vasculature through a catheter positioned near the target region. After release from the catheter, their movement is controlled toward the desired branch under real-time X-ray imaging guidance (Figure 1b). After reaching the target region, the microstructures can remain at the selected location and locally release the loaded therapeutic agents (Figure 1c).

## 2. Materials and Methods

### 2.1. Materials and Fabrication

Magnetically actuated microrobotic microstructures were fabricated using sodium alginate as a natural polymer and calcium chloride (CaCl_2_) as a crosslinking agent (Sigma-Aldrich, St. Louis, MO, USA). MNPs(Fe_3_O_4_, 50–100 nm; Sigma-Aldrich, St. Louis, MO, USA) were incorporated to provide magnetic responsiveness and X-ray contrast.

To optimize real-time intravascular visualization and precise magnetic actuation, microstructures with varying MNP concentrations were prepared. A 6% (*w*/*v*) alginate aqueous solution was prepared. MNP aqueous solutions at concentrations of 20%, 50%, and 100% were mixed with the alginate solution at a 1:1 volume ratio, yielding alginate–MNP mixtures with final MNP contents of 10%, 25%, and 50%, respectively. To prevent nanoparticle aggregation and ensure homogeneous dispersion within the hydrogel matrix, a combination of ultrasonication and vortex mixing was employed.

A 10% (*w*/*v*) CaCl_2_ aqueous solution was prepared in a conical tube, and the alginate–MNP mixture was dispensed into the CaCl_2_ solution as fine droplets using a custom-designed syringe–tube adapter while the tube was rotated at 800 RPM in a centrifuge. The resulting droplets underwent ionic crosslinking through Ca^2+^–alginate interactions and were gelled for 3 min to form spherical hydrogel micro-robotic microstructures. The fabricated microstructures were washed at least four times with deionized water (DW) and stored in DW until subsequent drug-loading and actuation experiments.

### 2.2. Characterization of Microstructures

The physical characteristics of the microstructures were examined using optical microscopy (Eclipse Ti-E, Nikon, Tokyo, Japan) and scanning electron microscopy (SEM; SU8010, Hitachi, Tokyo, Japan) to observe their overall morphology and surface microstructures. The spatial distribution of internal components was analyzed by energy-dispersive X-ray spectroscopy (EDX). Chemical bonding characteristics and thermal stability were evaluated using Fourier transform infrared spectroscopy (FT-IR; Perkin Elmer FT-IR Spectrum Two, Waltham, MA, USA) and thermogravimetric analysis (TGA; NE-TZSCH-Gerätebau GmbH, Selb, Germany), respectively. FT-IR spectra were acquired over the range of 400–4000 cm^−1^. TGA was performed from room temperature to 1000 °C to determine the compositional content of the microstructures quantitatively. In addition, magnetic hysteresis curves were measured using a vibrating sample magnetometer (VSM; Lake Shore Cryotronics 8604, Westerville, OH, USA, NFEC-2025-11-309744), from which the saturation magnetization values were obtained.

### 2.3. Therapeutic Agent Loading and Release

Doxorubicin (DOX) served as a model therapeutic agent to assess the drug-loading capacity of the microstructures. Microstructures containing varying concentrations of MNPs (10%, 25%, and 50%) were separately immersed in DOX solutions to facilitate drug loading through physical adsorption. Subsequently, the micro-structures were washed with DW to eliminate residual, non-adsorbed DOX. Prior to quantitative UV–vis absorbance analysis, the presence of loaded DOX was confirmed qualitatively by optical and fluorescence microscopy. The amount of drug loaded and the release profiles were determined quantitatively using UV–vis absorbance measurements, with a calibration curve generated from DOX standard solutions. Drug release was evaluated by immersing the DOX-loaded microstructures in DW as the release medium and measuring the absorbance of the re-lease solution at specified time intervals.

### 2.4. X-Ray Contrast Evaluation

The X-ray contrast performance of the microstructures was evaluated based on variations in image contrast corresponding to different concentrations of magnetic nanoparti-cles (MNPs) (10%, 25%, and 50%). Microstructures with different MNP concentrations were imaged under identical placement and acquisition conditions using a laboratory-designed X-ray imaging system operated at 100 kV with a maximum tube current of 100 μA. Following image acquisition, the radiopacity and contrast intensity of the microstruc-tures were analyzed and compared across conditions to assess the concentration-dependent X-ray contrast performance.

### 2.5. Magnetic Mobility and Control

The magnetically actuated microstructures are aligned and propelled within a three-dimensional workspace by time-varying magnetic fields generated by an EMA system. When a microstructure is exposed to an external magnetic field B, the resulting magnetic torque ($\tau_{mag}$) and magnetic force ($F_{m}$) are exerted to the microstructure as follows:

(1) τ=VM×B

(2) $$F_{m}=V\left(M\cdot \nabla \right)B$$

The volume, magnetization, flux density, and gradient of the microstructure are represented by V, $M\in R^{3\times 1}$, $B\in R^{3\times 1}$ and ∇, respectively.

Two types of EMA system configurations were implemented based on the underlying magnetic actuation principles. The first configuration utilized six orthogonally arranged rectangular electromagnetic coils to generate rotating magnetic fields within a three-dimensional workspace. Phase-shifted currents applied to the coils produced a rotating magnetic field, which induced rolling motion of the microstructures through magnetic torque alignment, as described by Equation (1). In the presence of the rotating magnetic field, the embedded MNPs within the microstructure continuously aligned with the field direction, generating a sustained rotational driving torque that enabled rolling-based propulsion [19]. This configuration was primarily used to evaluate the rotational mobility and rolling motion characteristics of the microstructures. In contrast, the second EMA configuration employed two electromagnetic coils with iron cores, designed to generate strong magnetic field gradients along a predefined axis. In this setup, spatial magnetic field gradients produced an attractive magnetic force on the microstructures according to Equation (2), enabling gradient-based pulling motion toward the target location. This configuration was used to assess translational guidance and positioning performance based on magnetic force–driven actuation.

The time-dependent magnetic field used for the rotating-field-based EMA configuration can be expressed in component form as:

(3) $$Β=\;\left[\begin{array}{c} B_{X}\left(t\right)\\ B_{y}\left(t\right)\\ B_{z}\left(t\right) \end{array}\right]=\;\left[\begin{array}{c} B_{0},rcos\theta cos\omega t\\ B_{0,}sin\theta cos\omega t\\ B_{0},rsin\omega t \end{array}\right]$$

where $B_{0,r}$ is the magnitude of the rotating magnetic field, ω is the angular frequency, and θ is an angular parameter that determines the field components.

In a fluidic environment, steady-state motion of the microstructure is achieved when the magnetic torque, $\tau_{mag},$ is balanced by the drag torque arising from viscous resistance, $\tau_{drag}$, which can be approximated as:

(4) $$\tau_{drag}=\zeta \;\omega$$

where ζ denotes the rotational friction (or viscous drag) coefficient. When the actuation frequency exceeds a critical threshold, known as the step-out frequency. This desynchronization causes phase lag and slip, which in turn produces a nonlinear decrease in propulsion speed.

During experimental evaluation with two types of EMA system configurations, microstructures were guided to target positions through precise control of the magnetic fields produced by the EMA systems. In both configurations, the currents supplied to each electromagnetic coil were independently regulated to generate specific magnetic field profiles for aligning and propelling the microstructures. Each coil’s current was managed by a motor driver controller (ESCON 70/10, Maxon Motor, Sachseln, Switzerland), which enabled accurate and real-time current control. Modulation of the amplitude and phase of the applied currents produced programmable magnetic field patterns, facilitating controlled guidance and movement of the microstructures under various experimental conditions.

To investigate the feasibility of intravascular magnetic guidance, a branched vascular phantom was fabricated using a 3D printer [20], in which the geometry was designed to reflect distal branch access conditions of a clinically used 1.4 Fr microcatheter (outer diameter: 0.57 mm), with the diameter of the main vessel set to approximately 4.1 mm. Flow within the phantom was generated by injecting PBS at a constant flow rate using a syringe pump, and the behavior of the microstructures was observed under flowing conditions. Magnetic guidance experiments were performed using an EMA system composed of two electromagnetic coils, where the direction and magnitude of the magnetic field within the phantom were controlled by regulating the currents applied to the coils. Branch-selection performance was quantified using the branch-selection efficiency (%), which was calculated as the percentage of microstructures entering the intended branch relative to the total number of microstructures reaching the bifurcation.

## 3. Results

### 3.1. Fabrication and Characterization of Magnetically Actuated Microrobotic Microstructures

Alginate-based hydrogel microstructures containing magnetic nanoparticles (MNPs) were fabricated by injecting Alginate–MNPs mixed solutions as fine droplets into a CaCl_2_ aqueous solution using centrifugal force. The microstructure size was primarily determined by the centrifuge’s rotational speed, which was maintained at 800 rpm in this study. To optimize intravascular maneuverability and real-time X-ray contrast performance, the MNP concentration was adjusted to 10%, 25%, and 50%, and the resulting microstructures were characterized through optical imaging. The centrifugal droplet generation process introduced the Alginate–MNPs mixtures into the CaCl_2_ solution as microscale droplets, which were subsequently crosslinked to form spherical hydrogel beads (Figure 2a).

Optical microscopy revealed that all microstructures retained a spherical morphology across MNP concentrations ranging from 10% to 50% (Figure 2b). Measurements of average diameter indicated that changes in MNP concentration had minimal impact on microstructure size, with all samples displaying a uniform size distribution of approximately 400 μm. These dimensions were designed considering the minimum diameter of standard angiographic catheters (approximately 2.0–2.4 French) [21], aiming for their applicability as a targeted drug delivery platform capable of navigating within the in vivo vascular environment (Figure 2c). SEM and EDX were conducted to further assess surface morphology and internal composition (Figure 2d). SEM images indicated that MNPs were evenly distributed within the alginate matrix without observable aggregation. Elemental mapping by EDX showed a marked increase in Fe signal intensity in the 25% and 50% MNP samples compared to the 10% condition, while Fe signal levels were similar between the 25% and 50% samples (Figure S1).

Magnetic characterization of the microstructures using a VSM confirmed superparamagnetic behavior in all samples, as indicated by the absence of magnetic hysteresis (Figure 2e). The saturation magnetization increased with higher MNP concentration, with the 25% and 50% samples exhibiting greater values than the 10% sample; however, no significant difference was observed between the 25% and 50% samples. TGA was performed to assess thermal stability and quantitatively analyze compositional content. The results demonstrated that alginate was fully decomposed at approximately 800 °C, leaving only residual MNPs (Figure 2f). The measured residual mass fractions for each sample closely corresponded to the initial mixing ratios of 10%, 25%, and 50%, confirming the compositional accuracy and reproducibility of the fabrication process. FT-IR spectra identified characteristic functional groups of both alginate and MNPs at all concentrations, supporting the formation of a stable hybrid microstructure without evidence of chemical modification or degradation of the constituent materials (Figure 2g).

### 3.2. Therapeutic Loading and Release Characteristics of Microstructures for Targeted Delivery

The effect of MNP concentration on drug loading capacity and release behavior was evaluated by loading microstructures containing 10%, 25%, and 50% MNPs with DOX. Optical and fluorescence microscopy confirmed successful DOX loading in all microstructures (Figure 3a). Quantitative analysis revealed maximum DOX loading amounts of 60.9 μg, 73.5 μg, and 75.6 μg for the 10%, 25%, and 50% MNP concentrations, respectively (Figure 3b). These results indicate an increasing trend in the loading capacity as the MNP concentration increases, which can be attributed to the interactions driven by surface functional groups (e.g., -COOH, -OH) generated upon mixing the natural polymer-based alginate matrix with the MNPs [22]. Drug release profiles were assessed by immersing DOX-loaded microstructures in PBS and measuring cumulative release over 72 h. After 3 h, cumulative release rates were 70.13%, 73.64%, and 54.76% for the 10%, 25%, and 50% MNP conditions, respectively, increasing to 91.34%, 92.00%, and 75.13% after 72 h (Figure 3c). Microstructures with 50% MNPs exhibited lower cumulative release at both early and extended time points. These results indicate that adjusting the MNP concentration allows tuning of drug delivery characteristics, enabling higher loading capacity while controlling the release rate.

### 3.3. Evaluation of X-Ray Attenuation of the Microstructures

The X-ray visibility of the microstructures at varying MNP concentrations was assessed by normalized grayscale intensities of X-ray images acquired under identical conditions for each sample. All microstructures were fabricated with consistent hydrogel geometry, and no materials other than MNPs were contained that could affect radiographic contrast (Figure 4a). X-ray images of microstructures with different MNP concentrations were obtained, and their contrast levels were compared. Microstructures with a 10% MNP concentration were not clearly discernible in the X-ray images, whereas those with 25% or higher MNP concentrations demonstrated progressively increased contrast intensity as concentration increased (Figure 4b). A comparison with the grayscale intensities of DW (>106.87) and the X-ray contrast agent (>66.15) demonstrates that increased MNP content enhances X-ray contrast (Figure 4c). Therefore, under identical size conditions, the X-ray visibility of the microstructures was quantitatively confirmed to increase with increasing MNP concentration.

### 3.4. Frequency–Velocity Actuation Characteristics of the Microstructures

Before conducting targeted delivery experiments, the magnetic mobility of the microstructures was evaluated using an EMA system. This system controlled both the direction and magnitude of the magnetic field within the workspace via coils arranged along three orthogonal axes, generating a uniform magnetic field with a maximum strength of approximately 20 mT (Figure 5a). Mobility evaluation was performed using a rolling-based actuation mode, in which magnetic torque was induced by a rotating magnetic field rather than a gradient-based pulling scheme. Therefore, magnetic field strength (mT) and rotational frequency (Hz) were selected as the primary actuation control parameters.

The effect of MNP concentration (10%, 25%, and 50%) on locomotion performance was investigated by measuring the average translational velocity of the microstructures as the magnetic field strength was varied from 1 to 20 mT and the actuation frequency from 0.5 to 20 Hz. Analysis of frequency-dependent velocity profiles at each magnetic field strength demonstrated that, under a low magnetic field of 1 mT, the average velocity remained nearly constant at approximately 0.06 mm/s across all frequencies and MNP concentrations. This result suggests that insufficient magnetic torque was generated to sustain rolling motion under these conditions. In contrast, at a magnetic field strength of 10 mT and a frequency of 20 Hz, the average velocities of microstructures with 10%, 25%, and 50% MNPs increased to 0.53, 1.22, and 1.80 mm/s, respectively. These values further increased to 2.65, 4.02, and 4.31 mm/s under 20 mT and 20 Hz conditions (Figure 5b,c). Within the frequency range of 0.5–10 Hz, the average velocity increased with increasing frequency, while a reduction in velocity was observed at frequencies above 20 Hz. This reduction is attributed to the actuation frequency approaching the step-out frequency of the microstructures, beyond which synchronization between the rotating magnetic field and microstructure motion deteriorates, resulting in reduced propulsion efficiency. Under the highest magnetic field and frequency conditions, increasing the MNP concentration from 10% to 25% led to an approximate 51.7% increase in velocity, while increasing it from 10% to 50% resulted in an approximate 62.6% increase. These results indicate that a magnetic field strength of at least 10 mT and an actuation frequency below 10 Hz are required for stable and controllable motion. Furthermore, MNP concentration was verified as a key design parameter governing both actuation responsiveness and locomotion performance.

### 3.5. Targeted Navigation Control of the Microstructures in a Vascular Phantom

To evaluate targeted navigation performance under intravascular conditions, magnetic guidance experiments were conducted using an EMA system in a branched vascular phantom that mimics the human portal vein geometry (diameter ≈ 4.1 mm) under controlled flow conditions (Figure 6a). In addition, the microstructures moved toward the target branch at the bifurcation according to the direction of the applied magnetic field. This result confirms that the movement direction can be controlled in real time under flowing conditions (Figure 6b and Video S1, Supplementary Materials). Using microstructures containing 10% MNPs, targeting performance was first compared at minimum and maximum flow rates, where the branch-selection efficiency toward both the left and right branches reached 100% at a minimum flow rate of Qmin = 2 mL/min, and high targeting efficiency was maintained even at a maximum flow rate of Qmax = 6.2 mL/min (Figure 6c). The average left- and right- branch-selection efficiencies were compared across different MNP concentrations (10%, 25%, and 50%) under maximum flow conditions. (Figure 6d).

To evaluate the X-ray visibility and branch-selective movement under a magnetic field, real-time X-ray imaging was performed for the control, left-targeted, and right-targeted groups. The results showed that real-time X-ray tracking and magnetic control could be successfully combined (Figure 6e and Video S2, Supplementary Materials). The mean grayscale intensities were 168.845 for the control group, 133.897 for the left-targeted group, and 131.036 for the right-targeted group. The lower grayscale intensities in the targeted branches indicated higher radiopacity in the branches where the microstructures were delivered (Figure 6f). Although all X-ray images were obtained under the same MNP concentration and flow rate conditions (50% and Qmid = 5.2 mL/min), the grayscale intensity of the control group was approximately 1.27-fold higher than those of the targeted groups. This difference may be caused by the magnetic navigation and immobilization of the microstructures inside the phantom. During magnetic targeting, the microstructures accumulated in the selected branch, resulting in a stronger local X-ray contrast. In contrast, the microstructures in the control group were not immobilized and continuously moved through the phantom, resulting in a weaker radiographic contrast.

## 4. Discussion

This study demonstrates the feasibility of magnetically actuated alginate-based microstructures as a platform for intravascular targeted delivery. The proposed microstructures were designed by containing MNPs into a Ca^2+^-crosslinked alginate hydrogel matrix, enabling magnetic actuation under an EMA system while simultaneously providing X-ray visibility for positional identification and control. Such combined functionality suggests that, following catheter-based delivery during clinical procedures, the microstructures can be further integrated into image-guided feedback loops for continuous magnetic guidance and control.

SEM/EDX and VSM analyses indicate that MNPs are stably contained in the microstructures, providing the magnetic responsiveness required for stable magnetic actuation. The observation of comparable Fe distributions and saturation magnetization values for the 25% and 50% MNP conditions is attributable to the assessment of magnetic properties based on the dry mass (emu/g) of freeze-dried samples containing both alginate and MNPs. Thus, despite an increased MNP concentration, similar effective compositions and mass-normalized magnetic properties in the freeze-dried samples may lead to only minor differences in the measured magnetic indicators between the 25% and 50% conditions.

DOX loading on the microstructures was achieved at all tested MNP concentrations (10%, 25%, and 50%), with higher loading capacities observed at 25% and 50% compared to 10%. However, drug release studies demonstrated that, while cumulative release increased over time for all samples, the 50% MNP condition consistently exhibited a lower release profile during both initial and extended periods. This trend is likely due to the increased effective surface area and greater number of adsorption sites for DOX–MNP interactions at higher MNP concentrations, leading to a larger proportion of DOX adsorbed or bound to MNP surfaces rather than freely diffusing within the hydrogel matrix. There-fore, during the release phase, DOX transport is influenced not only by diffusion but also by desorption from MNP sur-faces and increased internal transport resistance within the microstructure, ultimately limiting the overall release rate [23]. Previous studies have also demonstrated that surface functionalization or hybridization strategies can be used to load DOX onto iron oxide–based magnetic nanoparticle platforms [24,25], supporting the conclusion that interactions between iron oxide MNPs and DOX are critical for determining drug loading and release behavior.

X-ray visibility assessments revealed that microstructures containing 10% MNP were not clearly distinguishable in X-ray images. In contrast, microstructures with MNP concentrations of 25% or higher demonstrated substantially enhanced contrast. Thus, for X-ray–based localization and real-time tracking, MNP concentrations of 25% or higher are recommended. These results are based on visibility evaluations conducted in a phantom under controlled imaging conditions. Additional factors, such as tissue X-ray attenuation and hemodynamic conditions, may influence in vivo performance. Therefore, further validation through animal studies is required to assess X-ray visibility and targeted delivery performance of the proposed microstructures under conditions that more closely resemble the clinical environment.

The proposed microstructures consistently maintained a stable spherical morphology and a uniform size of approximately 400 μm across all MNP concentrations, while achieving both X-ray visibility and EMA-based magnetic actuation. X-ray–based localization was significantly improved at MNP concentrations of 25% or higher, enabling real-time tracking. However, the similar magnetic characteristics observed for the 25% and 50% MNP conditions indicate that further in-creases in MNP concentration do not necessarily result in proportional enhancements in actuation performance. Actuation experiments demonstrated stable and controllable velocity regulation at magnetic field strengths of at least 10 mT and actuation frequencies below 10 Hz. Furthermore, drug-loading and release characteristics varied with MNP concentration, indicating that MNP content can be adjusted to satisfy specific application and therapeutic requirements.

## 5. Conclusions

This study presents a magnetically actuated hydrogel-based microrobotic structure with intrinsic X-ray visibility designed for intravascular targeted drug delivery. Microstructures fabricated with varying MNP concentrations demonstrated real-time, precise actuation and stable control under external magnetic fields generated by an EMA system. Enhanced X-ray visibility was achieved with increasing MNP concentration, eliminating the need for additional contrast agents. Targeted delivery experiments in a vascular phantom simulating the human portal vein (diameter approximately 4.1 mm) confirmed an average targeting efficiency exceeding 89%, with directional controllability maintained under flowing conditions. While the maximum loading capacity of DOX increased with higher MNP concentrations, a lower release was observed at the 50% MNP condition, indicating a trade-off between drug loading efficiency and release performance. These findings indicate that the proposed platform offers tunable design and performance characteristics, supporting the development of image-guided, high-precision intravascular drug delivery and microrobotic therapeutic technologies.

## Figures and Tables

**Figure 1:**
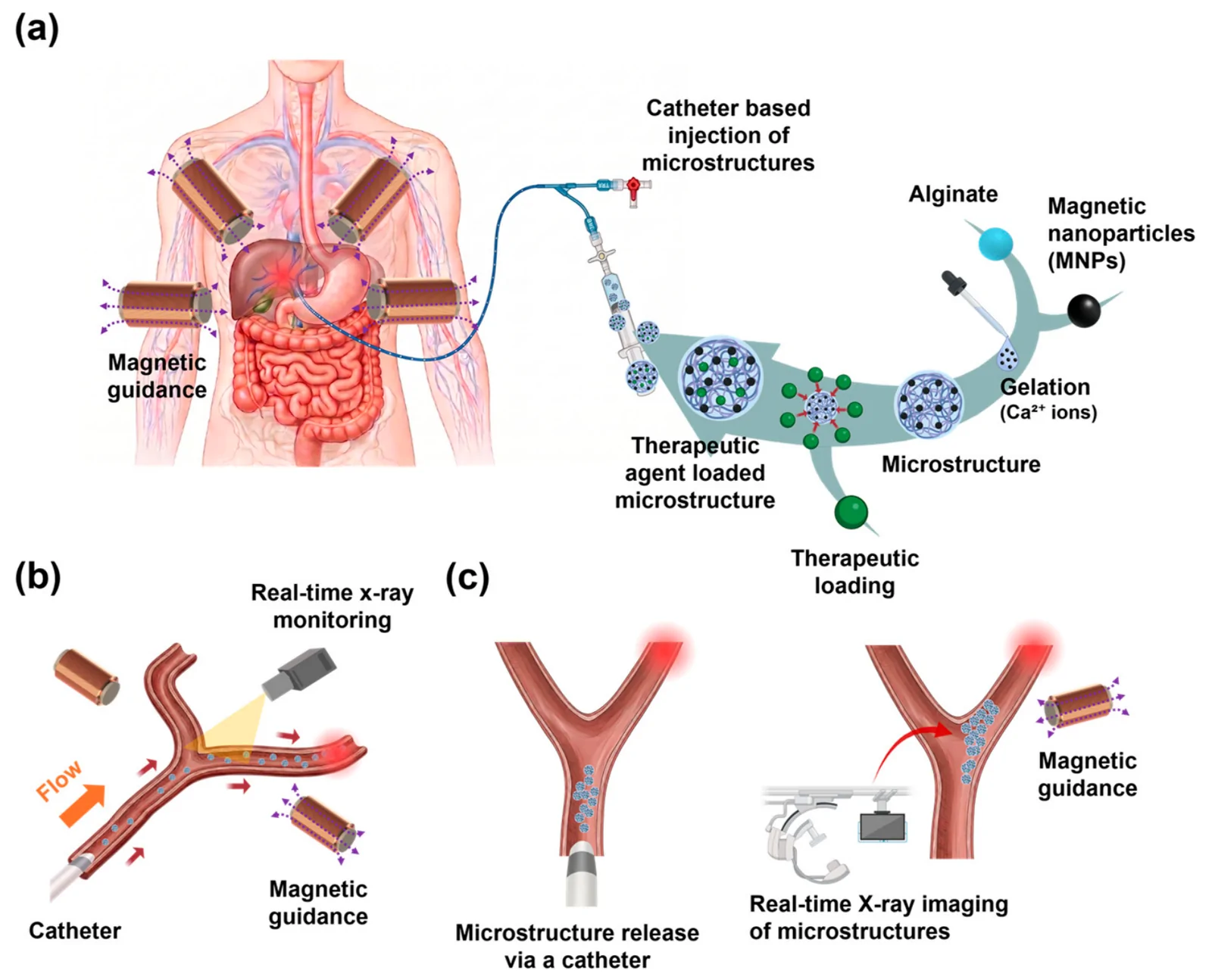
Conceptual illustration of vascular-based targeted therapy using magnetically actuated microrobotic microstructures. (**a**) Schematic illustration of a magnetically actuated microstructure composed of alginate-based hydrogel embedded with MNPs, and its catheter-based intravascular injection under control of an EMA system. (**b**) Illustration of real-time monitoring and magnetic guidance of the microstructures following catheter delivery under physiological flow conditions, where the red arrows indicate the microstructures moving along the magnetically induced trajectory. (**c**) Schematic depiction of the intravascular intervention process using the micro-structures, including release from a catheter, magnetic guidance toward the target site, and real-time tracking of the microstructures using live X-ray imaging.

**Figure 2:**
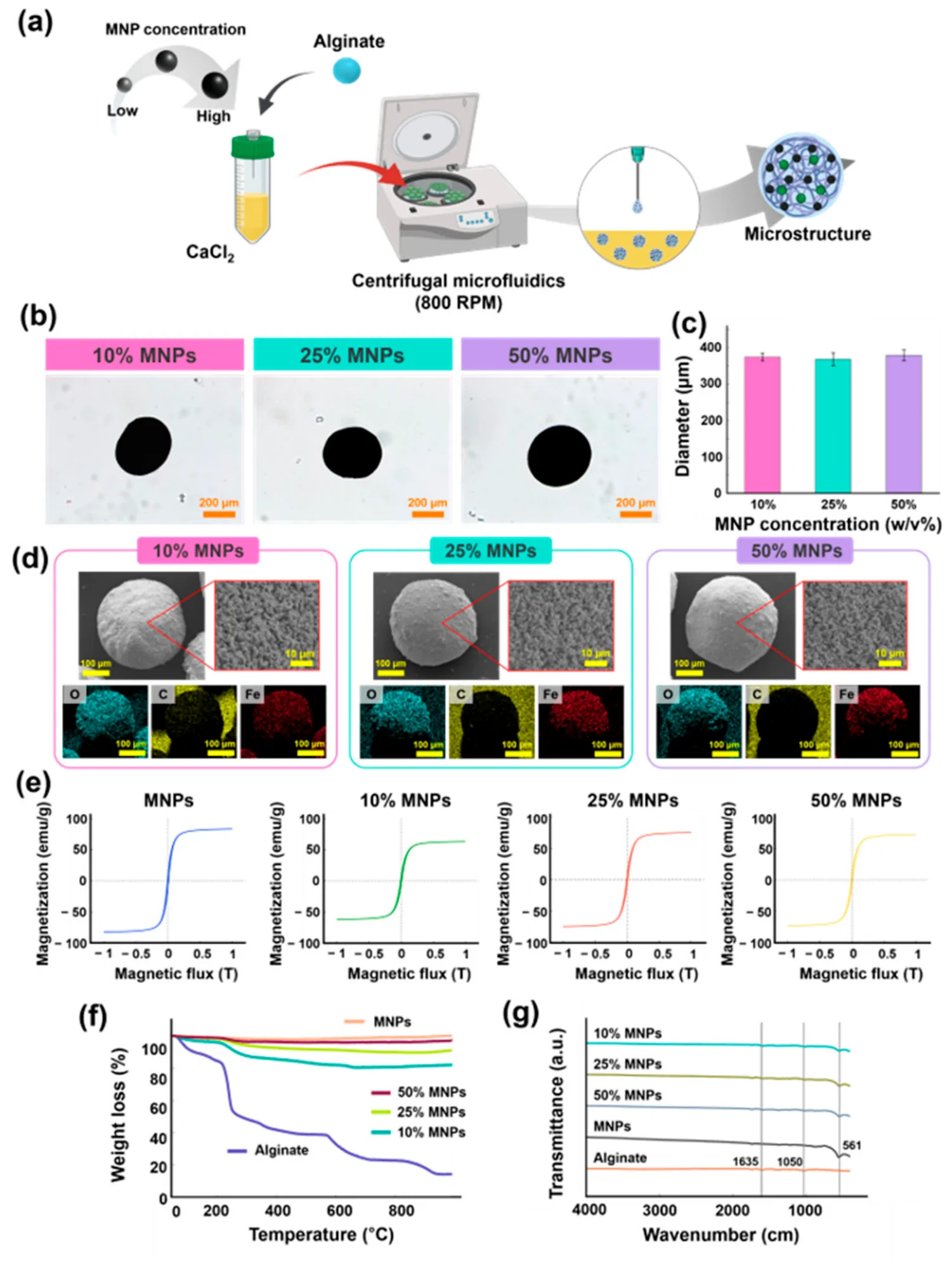
Fabrication and characterization of magnetically actuated microrobotic microstructures. (**a**) Schematic illustration of the fabrication process of alginate-based microstructures containing MNPs using a centrifugal microfluidic approach operated at 800 rpm. (**b**) Optical microscopy images of microstructures fabricated with different MNP concentrations (10%, 25%, and 50%). (**c**) Comparison of the average diameter of the microstructures as a function of MNP concentration. (**d**) SEM images and corresponding EDX elemental mapping of microstructures with MNP concentrations of 10%, 25%, and 50%, confirming the distribution of MNPs within the alginate matrix. (**e**) VSM hysteresis curves of pure MNPs and microstructures containing 10%, 25%, and 50% MNPs. (**f**) TGA results of alginate and microstructures with different MNP concentrations. (**g**) FT-IR spectra of alginate, MNPs, and microstructures containing 10%, 25%, and 50% MNPs.

**Figure 3:**
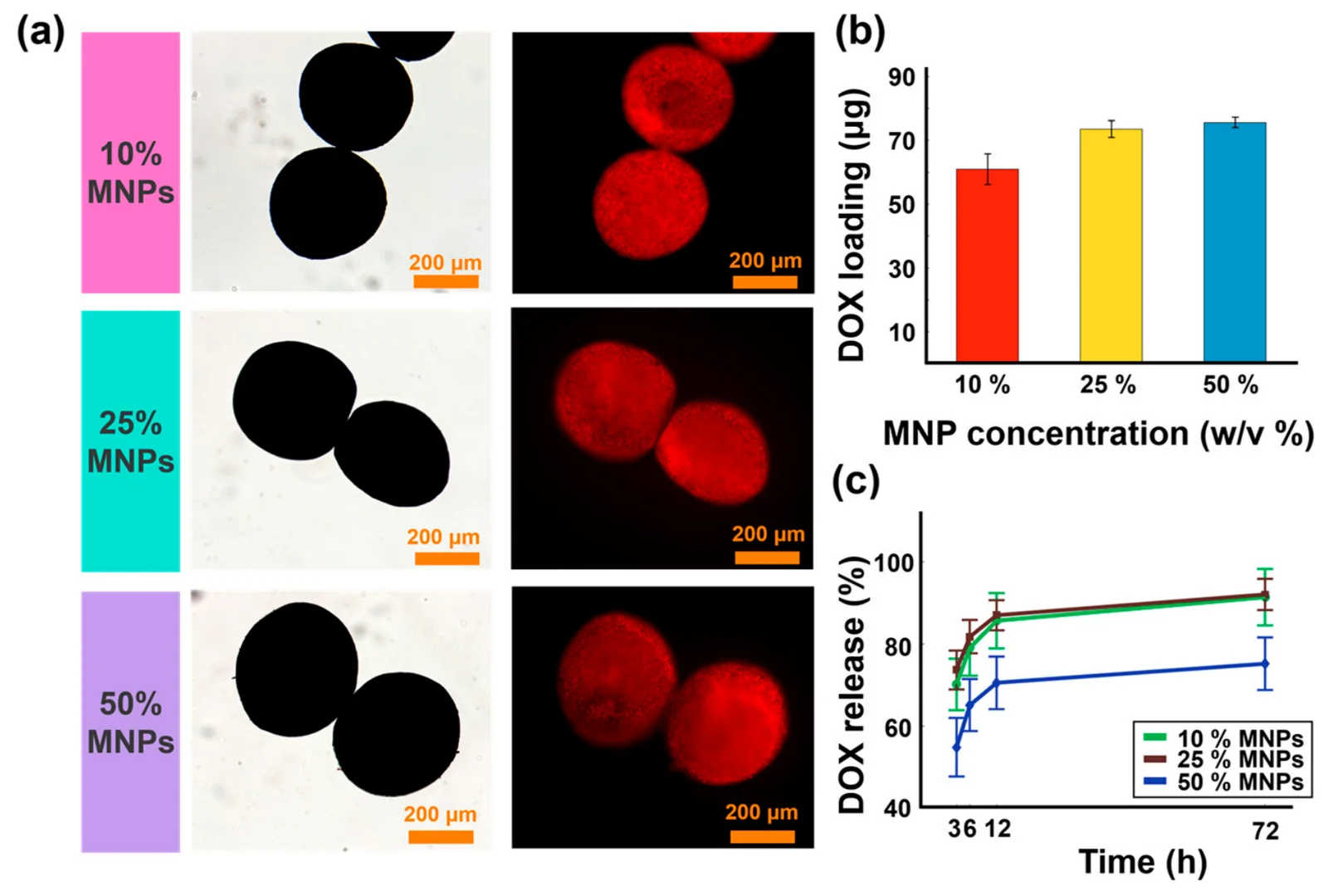
Drug loading and release characteristics of microrobotic microstructures for targeted delivery. (**a**) Optical (left) and fluorescence microscopy images (right) of the microstructures loaded with DOX at different MNP concentrations (10%, 25%, and 50%), where the red fluorescence confirms successful drug loading. (**b**) Maximum DOX loading amounts of the microstructures as a function of MNP concentration. (**c**) Cumulative DOX release profiles of the microstructures over time under aqueous conditions for different MNP concentrations.

**Figure 4:**
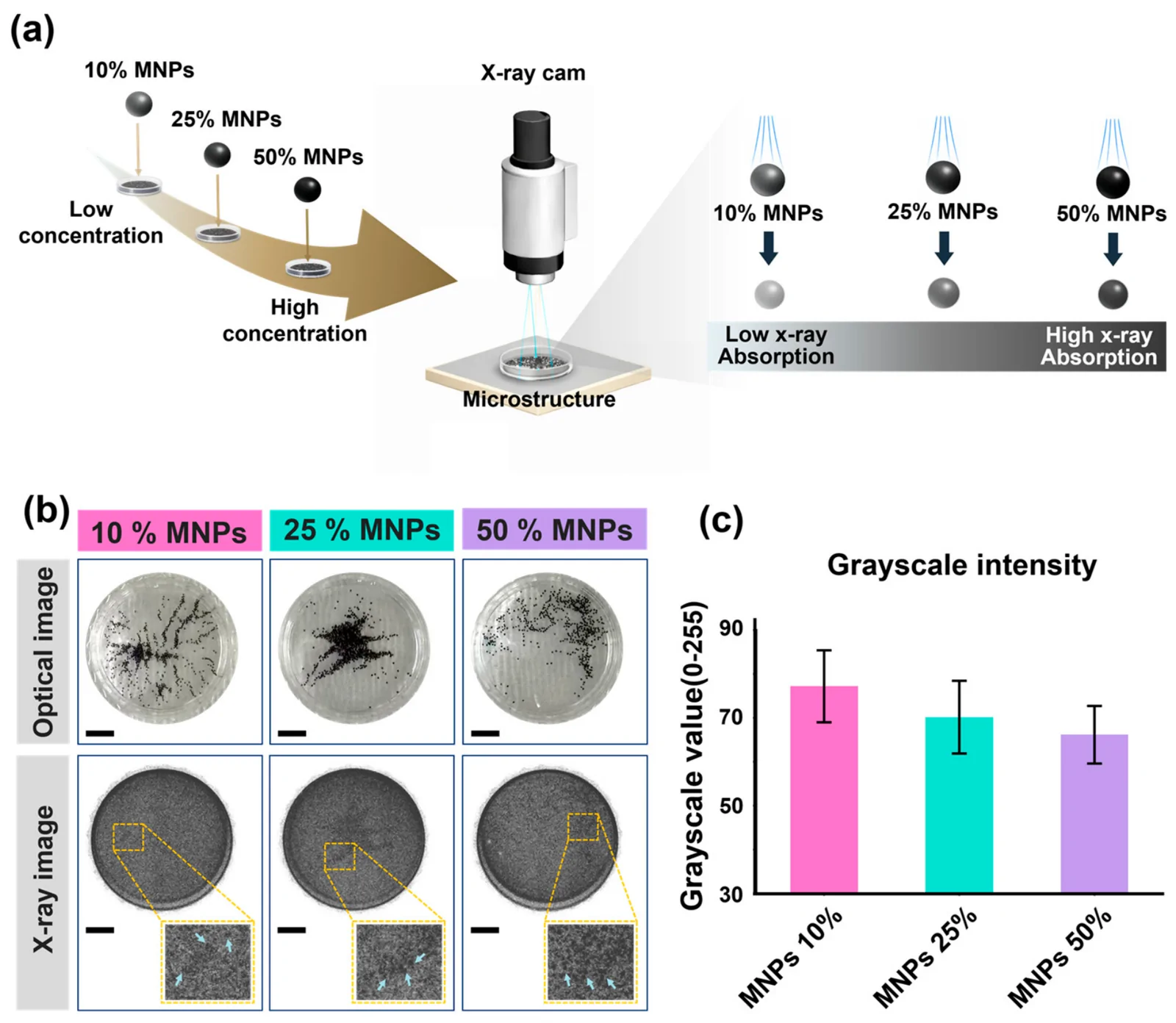
Evaluation of X-ray visibility of microrobotic microstructures as a function of MNP concentration. (**a**) Schematic illustration of the X-ray imaging setup and the X-ray visibility of the microstructures with increasing MNP concentration (10%, 25%, and 50%). (**b**) Optical images and corresponding X-ray images of microstructures with different MNP concentrations. The light blue arrows highlight the microstructures visible under X-ray imaging. Scale bars: 10 mm (**c**) Grayscale intensities of the targeted microstructures, demonstrating enhanced X-ray contrast with increasing MNP concentration.

**Figure 5:**
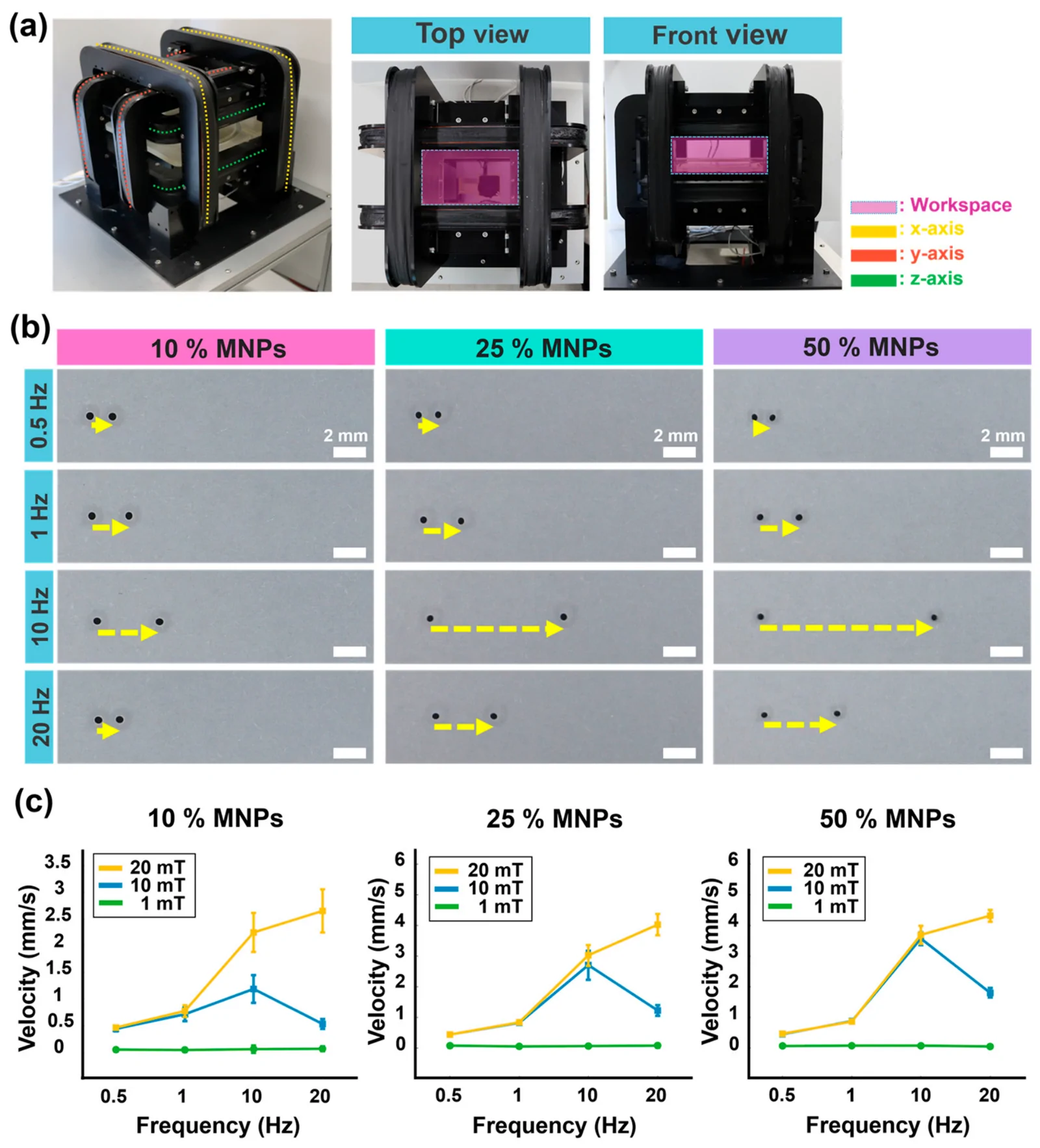
Frequency–velocity characteristics of microrobotic microstructures enabling precise control of magnetic locomotion. (**a**) Configuration of the EMA system that generates external magnetic fields to induce rolling motion in microstructures, with top and front views of the workspace. (**b**) Time-lapse images showing translational motion of microstructures with different MNP concentrations (10%, 25%, and 50%) under identical magnetic field strength (10 mT) and varying actuation frequencies (0.5, 1, 10, and 20 Hz), where the displacement over 3 s is indicated. The lengths of the yellow arrows represent this movement distance. (**c**) Average velocity of microstructures as a function of actuation frequency for various MNP concentrations at magnetic field strengths of 1, 10, and 20 mT.

**Figure 6:**
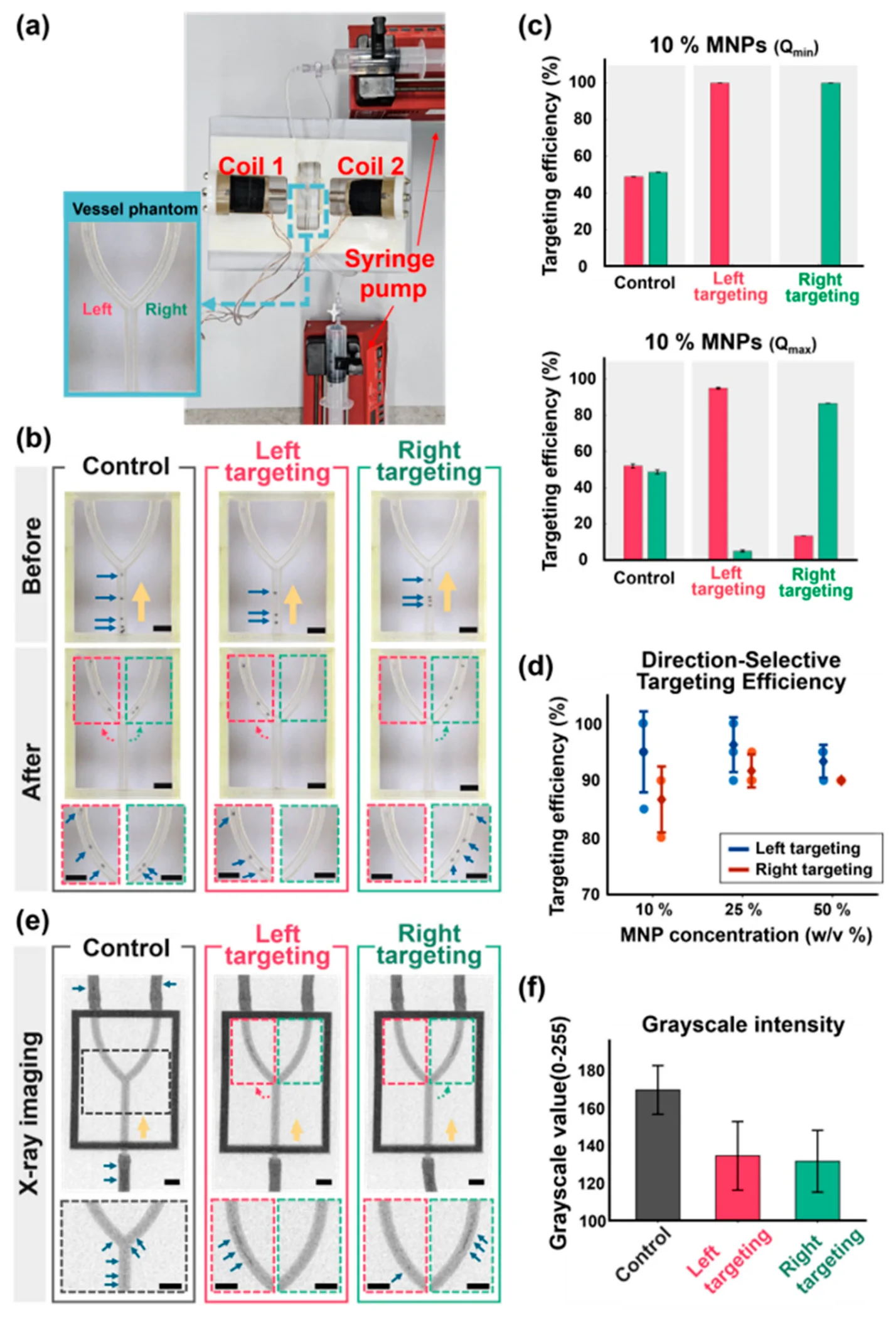
Real-time directional navigation control of microrobotic microstructures in a branched vascular phantom for intravascular applications. (**a**) Experimental setup showing the branched vascular phantom integrated with a syringe pump and a two-coil EMA system for magnetic guidance under flow conditions, with an inset image of the vessel phantom indicating left and right branches. (**b**) Representative optical images showing the directional movement of the microstructure before and after magnetic guidance into the left or right branch of a vascular bifurcation under various flow conditions (*n*> 3; Qmin = 2 mL/min, Qmax = 6.2 mL/min). Scale bars: 2 mm. (**c**) Branch-selection efficiency of microstructures containing 10% MNPs under minimal (*n* > 3, Qmin = 2 mL/min) and maximal (*n* > 3, Qmax = 6.2 mL/min) flow rates, comparing control, left- and right-targeting conditions (*n* > 3). (**d**) Left and right directional targeting efficiencies as a function of MNP concentration (10%, 25%, and 50%) under the maximum flow rate (*n* > 3). The dots represent individual data points, while the squares indicate the mean values. (**e**) Representative real-time X-ray images demonstrating the directional movement of the control, left- and right-targeted groups at a 50% MNP concentration and a flow rate (Qmid = 5.2 mL/min). The orange arrows denote the flow direction. The pink, and green arrows indicate targeting direction of the microstructures, respectively, while the navy arrows highlight the positions of the microstructures at the inlet and outlet of the phantom. Scale bar: 5 mm. (**f**) Grayscale intensities of the targeted microstructures under control, left- and right-targeting conditions.

## Data Availability

The original contributions presented in this study are included in the article/supplementary material. Further inquiries can be directed to the corresponding authors.

## Supplementary Materials

The following supporting information can be downloaded at https://www.mdpi.com/article/10.3390/mi17101120/s1, Figure S1: SEM–EDS zoom-in images of microstructures with different MNP concentrations; Video S1: Targeting tests of the magnetic actuated microstructures in a human vascular phantom; Video S2: Magnetic targeting of X-ray-visible microstructures in a human vascular phantom under real-time X-ray imaging.
