# Supplementary material for: Magnetically Actuated Hydrogel-Based Microrobotic Structures with X-Ray Visibility for Targeted Drug Delivery in Vascular Environments
Source: Micromachines (Basel). 2026 Sep 25;17(10):1120. doi: 10.3390/mi17101120 (PMC13706811; doi:10.3390/mi17101120)
Supplement: Supplementary file 1 [file micromachines-17-01120-s001.zip › Supplementary materials.pdf]

Supplementary Materials

Video Clips

Video S1. Targeting tests of the magnetic actuated microstructures in a human vascular phantom

Video S2. Magnetic targeting of X-ray-visible microstructures in a human vascular phantom under real-time X-ray imaging.

Figures

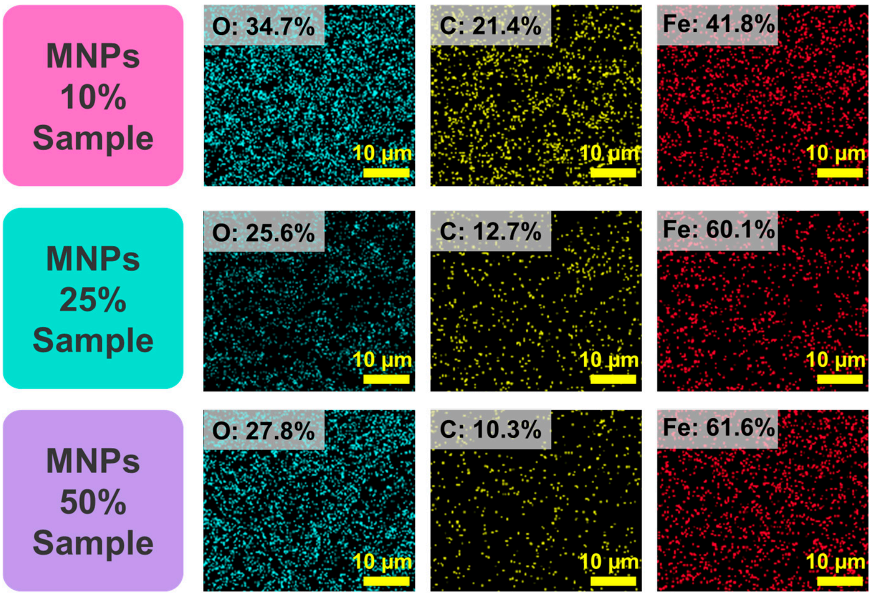

Figure S1. SEM–EDS zoom-in images of microstructures with different MNP concentrations.
